# Transcriptional Response of *Musca domestica* Larvae to Bacterial Infection

**DOI:** 10.1371/journal.pone.0104867

**Published:** 2014-08-19

**Authors:** Ting Tang, Xiang Li, Xue Yang, Xue Yu, Jianhui Wang, Fengsong Liu, Dawei Huang

**Affiliations:** 1 College of Life Sciences, Hebei University, Baoding, China; 2 Department of Pathology, Yale University, New Haven, Connecticut, United States of America; 3 Institute of Zoology, Chinese Academy of Sciences, Beijing, China; Uppsala University, Sweden

## Abstract

The house fly *Musca domestica*, a cosmopolitan dipteran insect, is a significant vector for human and animal bacterial pathogens, but little is known about its immune response to these pathogens. To address this issue, we inoculated the larvae with a mixture of *Escherichia coli* and *Staphylococcus aureus* and profiled the transcriptome 6, 24, and 48 h thereafter. Many genes known to controlling innate immunity in insects were induced following infection, including genes encoding pattern recognition proteins (*PGRP*s), various components of the Toll and IMD signaling pathways and of the proPO-activating and redox systems, and multiple antimicrobial peptides. Interestingly, we also uncovered a large set of novel immune response genes including two broad-spectrum antimicrobial peptides (*muscin* and *domesticin*), which might have evolved to adapt to house-fly's unique ecological environments. Finally, genes mediating oxidative phosphorylation were repressed at 48 h post-infection, suggesting disruption of energy homeostasis and mitochondrial function at the late stages of infection. Collectively, our data reveal dynamic changes in gene expression following bacterial infection in the house fly, paving the way for future in-depth analysis of *M. domestica*'s immune system.

## Introduction

Although lacking acquired immune systems, insects have efficient and potent innate immune systems to discriminate and combat foreign invaders successfully [1], [2]. It is generally acknowledged that the insect immune system involves cellular and humoral immune reactions against microbial infections that maintain close networks with each other and occur first in the epidermis, gut and tracheal respiratory organs and then in the hemocoel [2]. One characteristic of insect immunity is rapid activation of immune genes upon microbial infection, which produces effectors such as antimicrobial peptides. Insects have evolved sensitive mechanisms for recognition of pathogens including bacteria, fungi, parasites and viruses, which subsequently trigger cellular immune [3], [4] and humoral immune reactions [5], [6] via signal transduction pathways. Four signal transduction pathways, Toll, IMD, JNK and JAK/STAT, are regarded as the main pathways regulating the immune response of insects [7]. Genes encoding effectors are activated through signaling cascades and a set of these molecules are produced in specific tissues and secreted into the hemolymph [1].

House flies *Musca domestica* are endemic, and are the carriers of more than 100 harmful pathogens that can have severe consequences for human and animal health. Unfortunately, controlling the human diseases transmitted by house flies has not been successful due to the lack of knowledge of the basic molecular mechanism of this species [8]. Adaptation to distinct ecological environments might result in the evolution of specific immunity of house flies. Therefore, comparing the innate immune systems of *Musca* with those of the species that face different ecological pressures and pathogens such as *Drosophila* and *Anopheles* can be very informative, and thus offer clues on how house flies can flourish in close contact with many pathogens [8].

Recently, next generation sequencing technologies, such as the 454 Life Sciences (Roche) pyrosequencing platform, the Illumina Genome Analyzer, and the Applied Biosystems Solid platform provide rapid and high-throughput methods of identifying differentially expressed genes and their expression profiles [9], [10]. Identification and characterization of the host genetic factors released in response to pathogens is essential for understanding of innate immunity of *M. domestica*. However, information on the host genes involved in antibacterial defense is still limited. In this study, we performed transcriptome analysis and digital gene expression profile analysis of *M. domestica* challenged with *Escherichia coli* and *Staphylococcus aureus*, using high-throughput sequencing methods (Illumina Solexa Sequencing). The aims of this study were to uncover some information about the house fly immune response and discover new genes involved in bacterial infection in order to better understand the bacteria-host interaction. At the same time, the high-throughput sequencing in this study will identify a large number of transcripts that are comparable to the available transcripts in other species, and provide strong support for the genomic analysis of *M. domestica*.

## Materials and Methods

### Fly maintenance and bacterial challenge experiments

The laboratory colony of *M. domestica* used in this study was a gift from Miss Fengqin He, Institute of Zoology, Chinese Academy of Sciences. *Musca* larvae were raised on artificial diet consisting of bran and water until pupariation. After eclosion, adult flies were fed with water, sugar, and milk powder. Specimens at all life stages were kept in a temperature-controlled room at 25±1°C, 70±5% relative humidity, and a photoperiod cycle of LD12:12. Septic injury was produced by pricking the abdomen of the 2^nd^ instar larvae with a needle previously dipped into a concentrated mixed bacteria suspension of *E. coli* and *S. aureus* [11]. The bacterial challenged larvae were maintained at 25°C on fresh medium for 6 h, 24 h, and 48 h before RNA extraction.

### RNA isolation

Total RNA was isolated from the following developmental stages: eggs, 1^st^ instar larvae, 2^nd^ instar larvae, 3^rd^ instar larvae, pupae and newly-emerged adults (within 3 days of eclosion) in a 1∶1 female∶male ratio. RNA was extracted using the RNAiso Plus Kit (TaKaRa) following the manufacturer's instructions.

### Construction of the cDNA library and Illumina sequencing for transcriptome analysis

Briefly, 20 mg total RNA (a mixture of RNA from eggs, 1^st^ instar larvae, 2^nd^ instar larvae, 3^rd^ instar larvae, pupae, adults, and bacterial challenged 2^nd^ instar larvae at equal ratios) was used to construct a cDNA library. Poly (A) mRNA was purified from total RNA using oligo (dT) magnetic beads. Fragmentation buffer was added for resizing mRNA to short fragments. Taking these short fragments as templates, random hexamer-primer was used to synthesize the first-strand cDNA. The second-strand cDNA was synthesized using buffer, dNTPs, RNaseH and DNA polymerase I, respectively. Short fragments were purified with the QiaQuick PCR extraction kit and resolved with EB buffer for end reparation and adding poly (A). After that, the short fragments were connected with sequencing adapters. And, after the agarose gel electrophoresis, the suitable fragments were selected for the PCR amplification as templates. Finally, the library could be sequenced using Illumina HiSeq™ 2000.

### Bioinformatics analysis

Transcriptome de novo assembly was carried out with short reads assembling program Trinity [12]. Trinity first combines reads with a certain length of overlap to form longer fragments, which are called contigs. The reads are then mapped back to contigs. The paired-end reads enable the software to detect contigs from the same transcript as well as the distances between these contigs. Next, Trinity connects the contigs, and gets the sequences that cannot be further extended on either end. Such sequences are defined as unigenes. The unigenes were lengthened by the increased sequence depth using normalized library with the 454 platform [13]. Finally, BLASTx alignment (E-value<10^−5^) between unigenes and protein databases like nr, Swiss-Prot, KEGG and COG (Clusters of Orthologous Groups of proteins) were performed, and the best aligning results were used to decide sequence direction of unigenes. When different databases gave conflicting results, we prioritized them in the following order: nr, then Swiss-Prot, KEGG, and COG. When unigene did not align with any of the entries in these databases, ESTScan [14] was used to predict the unigene's coding regions and to determine its sequence direction.

Candidates of immunity-related genes from the fruit fly were used to query the *M. domestica* transcriptome. The nucleotide CDS and protein amino acid sequence for each of the identified *Drosophila melanogaster* immune genes were downloaded from the flybase (http://flybase.org/). The *M. domestica* transcriptome was searched for homologues to these sequences using CLC Main Workbench 5.5 with the tBLASTn program with a cutoff E-value of 10^−5^. Tentative matches were checked by searching the nr NCBI database using BLASTn for gene prediction errors.

We collected the unigenes that are homologous to the important known innate-immunity-relevant genes, and aligned them to the reference gene sequences of *D. melanogaster* for counting the gene variant numbers. At first, we included the reference gene variants of a certain *D. melanogaster* gene together with the homologous unigenes from our data to do the alignment, and then we assigned the unigenes to their most homologous reference gene variants respectively. As for those none-full-length unigenes, sometimes we couldn't tell which reference gene variant they belong to because of their lacking of the specific sequence region, so that we assigned them to the first reference gene variant showed in the alignment result. Finally we counted the least variant number of the unigenes under each reference gene variant, and added up the numbers as the least variant gene number of the gene in the house fly. When we counted the least variant number of the unignenes, in order to avoid counting the none-overlapping fragments from the same variant, we only took into account the unigenes that overlap with each other and cover the highest phylogenetic diversity site in the alignment.

### Gene expression library preparation and sequencing

Total RNA was extracted separately from immune-challenged larvae at 6, 24 and 48 h post-infection following the manufacturer's instruction, as described above. RNA extracted from non-challenged larvae at each matching time point was taken as a control. Ten larvae were collected for RNA extraction from each group. Next, a gene expression library was prepared using an Illumina gene expression sample prep kit. Briefly, mRNA was enriched using the oligo (dT) magnetic beads from the total RNA, and cDNAs were synthesized as described above for each sample. The library products were then ready for sequencing analysis via Illumina HiSeq™ 2000 using paired-end technology in a single run. Six libraries from control and challenged groups were constructed.

### Analysis and annotation of gene expression tags

The 50 bp length reads were gained through base calling and were filtered to get the clean reads by removing the dirty reads with adaptors, reads in which unknown bases are more than 10%, and low quality reads (the percentage of the low quality bases of quality value ≤5 is more than 50% in a read). Then, clean reads were mapped to the above transcriptome reference database using SOAPaligner/soap2 [15], allowing no more than 2 mismatches. Gene expression levels were evaluated with RPKM values [16], and the value was substituted by 0.001 if the gene has no expression. NOIseq method that can work in absence of replication was used for gene expression analysis [17]. If there was more than one transcript for a given gene, the longest transcript was used to calculate its expression level and coverage. To identify differentially expressed genes between two samples, the false discovery rate (FDR) method was used to determine the threshold of P-value in multiple tests [18]. We use “FDR≤0.001 and the absolute value of log_2_Ratio ≥1” as the threshold to judge the significance of gene expression difference. In the gene expression profiling analysis, Gene Ontology (GO) enrichment analysis of functional significance was applied using the hypergeometric test to map all differentially expressed genes to terms in the GO database, looking for significantly enriched GO terms in differentially expressed genes, and comparing them to the transcriptome database. For the pathway enrichment analysis, we mapped all differentially expressed genes to terms in the KEGG database and looked for significantly enriched metabolic pathways or signal transduction pathways in differentially expressed genes comparing with the whole transcriptome database. Then, all genes expression was subjected to KEGG enrichment analysis compared to the transcriptome background using a hypergeometric test. Annotated KEGG pathways with a Q value≤0.05 were considered as significantly enriched terms in differentially expressed genes.

### qPCR validation

The accuracy of the genes expression approach has been validated by qPCR analysis. Total RNA was extracted from bacterial challenged and non-challenged larvae as described for gene expression library preparation, and then was reverse-transcribed according to the protocol provided with M-MLV reverse transcriptase (Promega, USA). Ten differentially expressed genes were randomly chosen to verify gene expression sequencing results using four replicates. The β-actin gene was used as a constitutive expression control for normalization. The primers are shown in Table S1. qPCR was performed following the above-mentioned methods with modified annealing temperature for each pair of primers [19]. The relative quantification (comparative method) was calculated using the ΔΔCt method [20]. All samples were normalized to the ΔCt value of a reference gene to obtain a ΔΔCt value (ΔCt target − ΔCt reference). The final relative expression was calculated using the following formula: F = 2^−ΔΔCt^. The data obtained from qPCR were analyzed for statistical significance using Graph-Pad Prism [21]. The significance at P<0.05 was analyzed using one-way ANOVA. The qPCR results were then compared with gene expression data to detect the expression correlation of each gene.

### Peptide synthesis and antibacterial assays

The peptides muscin and domesticin, were chemically synthesized using the solid-phase method on the Applied Biosystems model 430A peptide synthesizer by Shanghai mocell biotech Co., Ltd. (Shanghai, China). Synthesized peptides were purified by high performance liquid chromatograph (HPLC), and verified by mass spectrometry and amino acid composition analysis. Each synthesized peptide was diluted with sterile deionized water to different concentrations, and applied directly to antibacterial assays. Twelve bacterial strains used in the tests were a gift from Shunyi Zhu, Institute of Zoology, Chinese Academy of Sciences (Beijing, China). Minimum inhibitory concentrations (MICs) were determined in duplicate by the liquid growth inhibition assay [22]. Briefly, 10 µl of each dilution (sterile deionized water as a control) were incubated in sterile microtiter plates (96 wells) with 100 µl of a suspension of midlogarithmic phase culture of bacteria at a starting optical density of OD_600_ = 0.001, or with 80 µl of fungal spores (final concentration 10^4^ spores/ml) and 10 µl of water. Poor-broth nutrient medium (1% bactotryptone, 0.5% NaCl w/v, pH 7.5) was used for standard bacterial cultures. Anti-fungal assays were performed in potatoes dextrose broth (Difco) at half-strength supplemented with 10 mg/ml tetracycline. Bacteria were grown during 24 h under vigorous shaking at 30°C. Fungi were grown at 25°C in the dark without shaking for 48 h in a moist chamber. Microbial growth was controlled by measurement of the optical density at D_600_ after a-24 h incubation. Inhibition of filamentous fungi growth was observed at microscopic level after 24 h and measured at 600 nm after 48 h. The MIC values are expressed as the range between the highest concentration of the peptide where bacterial growth was observed and the lowest concentration that caused 100% of inhibition bacterial growth [23].

## Results and Discussion

### Sequencing and sequence assembly

To obtain the global gene expression profile of the house fly, RNA samples from *M. domestica* of six developmental stages (eggs, 1^st^ instar larvae, 2^nd^ instar larvae, 3^rd^ instar larvae, pupae, adults) were prepared, equally-mixed and then sequenced by the Illumina platform in a single run which generated 70,335,268 raw reads, 66,049,270 clean reads and 5,944,434,300 total clean nucleotides (Table 1). The average read size, Q20 percentage (sequencing 1% error rate,) and GC percentage were 90 bp, 96.25%, and 53.69%, respectively. These short reads were assembled into 116,687 contigs with a mean length of 319 bp. Then, after gap filling of contigs using paired-end reads from the transcriptome sequencing data of 454, we obtained 47,086 unigenes. The mean size of these unigenes was 757 bp and the N50 was 1,186 bp. Here N50 was the length of the smallest contig in the set that contained the fewest or largest contigs whose combined length represents at least 50% of the assembly. Of these unigenes, 10,933 (23.2%) were larger than 1,000 bp (Figure S1). Due to the short length of the sequencing read, unigenes obtained from Illumina platform were short in general. In present study, the unigenes were lengthened by the increased sequence depth using our previous normalized library with 454 platform [13].

**Table 1:** Summary of the *M. domestica* transcriptome.

Parameters	Number
Total clean reads	66,049,270
Total Nucleotides (nt)	5,944,434,300
Total number of contigs	116, 687
Total number of unigenes	47,086
Mean size of unigenes (bp)	757
N50 of unigenes (bp)	1,186
Sequences matched known genes with E-value<10^−5^	27,021

### Unigene functional annotation

Unigene sequences were annotated by searching the Nr of NCBI, Swiss-Prot, KEGG and COG protein database using BLASTx with a cut-off E-value of 10^−5^. A total of 27,021 distinct sequences (57.39% of unigenes) matched known genes (Table S2). The remaining 20,065 unigenes (42.61%) could not be done and needed more genetic data to annotate. It is noteworthy that a large part of the unigenes in *M. domestica* transcriptome database is with unknown functions.

**Figure 1:**
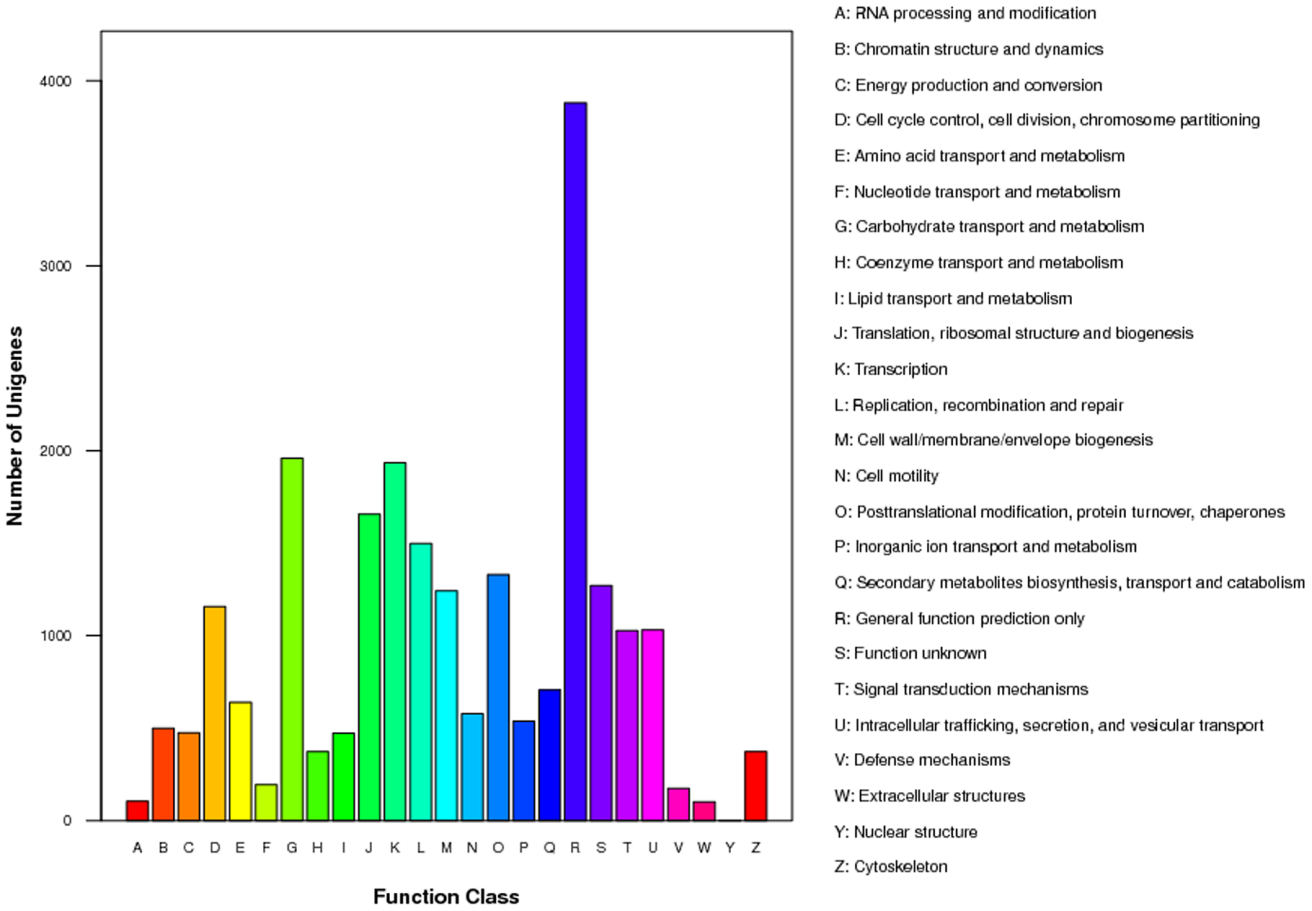
Classification of the clusters of orthologous groups (COG) for the *M. domestica* transcriptome. 8,549 unigenes (31.6% of the total annotated unigenes) were divided into 25 specific categories.

Based on sequence homology, 23,212 unigenes (85.9%) were annotated and divided into 25 different COG categories (Figure 1). The general function category that contained 3,880 unigenes (16.7%) was the largest, followed by carbohydrate transport and metabolism (1,959, 8.4%), transcription (1,935, 8.3%), translation, ribosomal structure, and biogenesis (1,656, 7.1%), eplication, recombination and repair (1,498, 6.5%), and post-translational modification, protein turnover, and chaperones (1,329, 5.7%). Only two unigenes belonged to nuclear structure, which was the smallest group.

**Figure 2:**
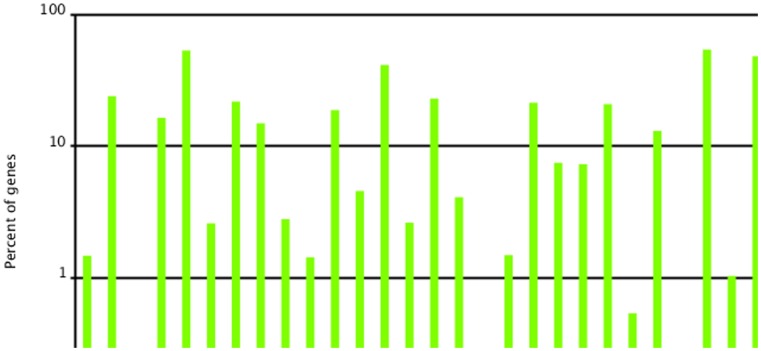
Classification of the gene ontology (GO) for the *M. domestica* transcriptome. 7,063 unigenes (26.1% of total) were categorized into 48 function groups.

The GO categories recovered from Blast2GO analyses were summarized by the proportion of unique sequences annotated in each GO level 2 category (Figure 2). We categorized 7,063 unigenes (26.1% of total) into 48 function groups. Cell, cellular process, cell part, binding and metabolic process were the five largest groups, containing 3,812, 3,751, 3,389, 2,919, and 2,914 unigenes, respectively.

To identify the biological pathways that are active in *M. domestica*, functional classification and metabolic pathway assignment were performed by the KEGG annotation system. A total of 16,440 unigenes (60.8%) were classified into 239 KEGG pathways. Metabolic pathways contained 2,274 unigenes (13.8%) was significantly larger than other pathways, such as pathways in cancer (619, 3.77%), focal adhesion (565, 3.44%), and regulation of actin cytoskeleton (545, 3.32%). In addition, some immune related pathways, including phagosome, lysosome, ECM-receptor interaction, Fc gamma R-mediated phagocytosis, leuko-cyte trans-endothelial migration, complement and coagulation cascades, and many signaling transduction pathways such as MAPK, VEGF, JAK-STAT, PPAR, and Toll-like receptor signaling pathway were predicted in the KEGG database (Table S3).

These annotations provide a valuable resource for investigating specific processes, functions and pathways and allow for the identification of novel genes involved in the pathways of immunity.

### Annotation of immune-relevant genes

Based on genome-wide analysis, many immune-related genes have been identified from *D. melanogaster* (265), *Anopheles gambiae* (304), *Apis mellifera* (138), and *Bombyx mori* (220) [2]. To gain deep insight into the molecular biology of immune systems in *M. domestica*, the immune-relevant genes were discovered by referencing to those identified in other insect species. Approximately 279 genes were found to be homologous to known immune-relevant genes (Table 2), including the most important elements of innate immunity, such as pattern recognition receptors, and other immune-related genes (such as *PPO*, *NOS*, *caspase*, *dicer*2, *argonaute*2). It is noteworthy that many unigenes obtained by next generation sequencing should be different fragments or even allelic or splice variants of the same gene [24]. In this study, the number of gene sequences predicted to encode immune genes would be therefore overestimated over the actual number of genes belonging to each of the currently characterized gene families. The putative genes uncovered provide the basis for further understanding of the physiological functions of candidate genes in *M. domestica* immune responses.

**Table 2:** Gene counts belonging to immunity-related gene families.

Gene family	Gene numbers				
	*Drosophila melanogaster* a	*Anopheles gambiae* a	*Apis mellifera* a	*Bombyx mori* a	*Musca domestica* b
**Recognition**					
*PGRP*	13	7	4	12	16
*βGRP*	3	7	2	4	3
*fibrinogen*-*related protein*	14	61	2	3	19
*scavenger receptor*	21	21	13	18	13
C-type *lectin*	34	25	10	21	16
*hemocytin*	1	0	1	1	1
*galectin*	6	8	2	4	7
*TEP*	6	15	3	3	36
*nimrod*	10	4	4	4	8
*draper*	1	1	1	1	2
*eater*	1	1	0	0	0
*dscam*	1	1	1	1	1
**Modulation**					
*clip serine protease*	37	41	18	15	6
*serpin*	30	17	5	26	40
**Toll pathway**					
*spätzle*	6	6	2	3	1
*toll*	9	10	5	14	14
*MyD*88	1	1	1	1	1
*tollip*	1	2	1	2	1
*tube*	1	1	1	1	1
*pellino*	1	1	1	1	1
*pelle*	1	1	1	1	1
*TRAF*2	1	1	1	1	0
*ECSIT*	1	1	1	1	2
*cactus*	1	1	3	1	2
*Dif/Dorsal*	2	1	2	1	2
**IMD pathway**					
*IMD*	1	1	1	1	1
*DREDD*	1	1	1	1	1
*TAK*1	1	1	1	1	1
*FADD*	1	1	1	1	1
*TAB*2	1	1	1	1	2
*IAP*2	1	1	1	1	1
*IKK*	2	2	2	2	1
*UBC*13	1	1	1	1	0
*relish*	1	1	2	1	1
**JNK pathway**					
*HEM*	1	1	1	1	1
*JNK*	1	1	1	1	3
*FOS*	1	1	1	1	1
*JUN*	1	1	1	1	1
**JAK/STAT pathway**					
*UPD*3	1	0	0	0	0
*PIAS*	1	1	1	1	1
*SOCS*	1	1	1	1	1
*domeless*	1	1	1	1	4
*hopscotch*	1	1	1	0	2
*STAT*	1	2	1	1	1
**Antimicrobial peptide**					
*ceropin*	4	4	0	13	3
*attacin*	4	1	0	2	9
*diptericin*	2	0	0	0	3
*defensin*	1	4	2	1	1
*gloverin*	0	0	0	4	0
*moricin*	0	0	0	9	0
*lebocin*	0	0	0	1	0
*domesticin* *	1	0	0	0	1
*muscin* *	0	0	0	0	1
**Melanization**					
*PPO*	3	9	1	2	7
*DDC*	1	7	1	1	2
*DCE*	2	0	18	16	4
*TH*	1	1	1	1	2
*punch*	1	0	1	0	1
**Other effectors**					
*NOS*	1	1	1	2	1
*POI*	2	1	0	1	0
*lysozyme*	11	8	2	4	13
**Other immune molecules**					
*caspase*	6	11	4	4	5
*dicer*2	1	1	1	0	6
*argonaute*2	1	1	1	1	2
Total	265	304	138	220	279

a Number of gene sequences from data of Christophides et al. (2002) [57], Evans et al. (2006) [7], Tanaka et al. (2008) [2].

b Number of gene sequences obtained in this study that annotated with NCBI nr database.

* Two novel antimicrobial peptides identified and named in present study.

### Gene expression library sequencing

Having established the house fly transcriptome database, we next determined how bacterial infection altered the transcriptome. We sequenced RNA from 2^nd^ instar larvae at 6 h, 24 h, 48 h following inoculation with a mixture of *E. coli* and *S. aureus*; RNA from uninfected larvae served as the control (designated as M6, M24, M48 and CK6, CK24, CK48, respectively). After filtering the dirty tags, each sample generated 3,510,021∼3,672,652 reads. Among these clean reads, 78.51∼86.51% were mapped to unigenes in the transcriptome database (Table 3). The percentage of clean reads ranged from 96.78% to 99.29%, reflecting the high quality of the sequencing. About 4∼5% of genes were covered between 90∼100% in each library.

**Table 3:** Statistics of gene expression sequencing.

Summary	M6	CK6	M24	CK24	M48	CK48
Total reads	3,661,408	3,510,021	3,513,957	3,661,248	3,560,749	3,672,652
Total base pairs	179,408,992	171,991,029	172,183,893	179,401,152	174,476,701	179,959,948
Total mapped reads	3,154,425	3,015,374	3,040,076	2,874,614	2,971,222	3,041,448
Perfect match	2,421,048	2,312,301	2,287,588	2,002,766	2,242,858	2,159,328
≤2 bp mismatch	733,377	703,073	752,488	871,848	728,364	882,120
Unique match	2,216,969	2,097,216	2,069,330	2,134,543	2,166,611	2,164,870
Multi-position match	937,456	920,759	970,746	740,071	804,611	876,578
Total unmapped reads	506,983	494,647	473,881	786,634	589,527	631,204

### Effects of bacterial infection on gene expression

**Figure 3:**
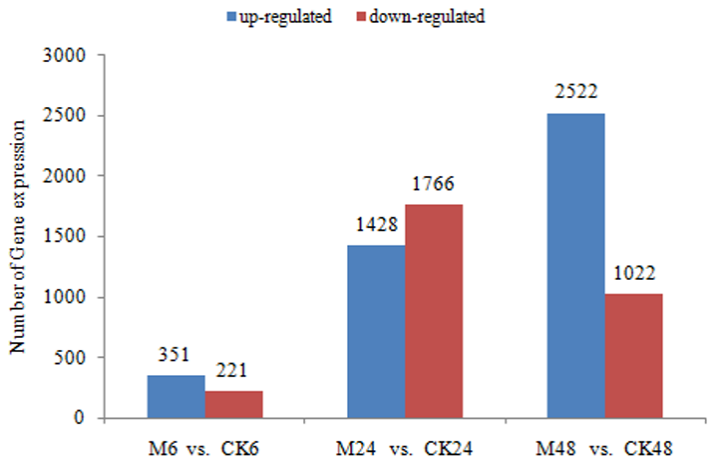
Differentially expressed genes of each library compared with CK. Unigenes changed at transcriptional level following bacterial challenge were identified by filtering of the one-fold up- and down-regulated ones with FDR≤0.001.

The “FDR≤0.001” and the absolute value of “log_2_ Ratio≥1 or ≤−1” were used as the threshold to identify and compare differentially expressed genes between larvae non-challenged and challenged at different stages. Numbers of differentially expressed genes for each comparison were shown in Figure 3. 572, 3194, and 3544 genes were significantly affected by bacterial infection at 6, 24 and 48 h post-infection, respectively (Table S4), They include genes involved in insect immunity (ECM-receptor interaction, phagosome, complement and coagulation cascades, PPAR signaling pathway) and protein, carbohydrate and lipid metabolism. Besides, genes reflecting mitochondrial oxidative stress and endoplasmic reticulum (ER) stress were up-regulated. Thus, infection caused extensive changes in the *M. domestica* physiological status.

Importantly, some of these changes are temporal-specific. There were 12, 33, 43 pathways significantly enriched in the 3 comparisons respectively (Q value≤0.05). The results are shown in Table S5. For example, at 6 h after bacterial infection, metabolic pathways were preferentially altered, with the genes affected including those controlling fatty acid, galactose and amino acid metabolism, ribosome biogenesis, phagocytosis, neuroactive ligand-receptor interaction, collecting duct acid secretion, terpenoid-quinone biosynthesis and adipocytokine signaling. In contrast, at later stages, particularly at 48 h post-bacterial challenge, genes controlling oxidative phosphorylation were strongly down-regulated, suggesting profound mitochondrial dysfunction at these stages.

### Differentially expressed genes involved in immune response

Among the genes affected by infection, 509 were presumably involved in innate immune responses (Table S6), including pattern recognition, Toll and IMD signaling, direct antimicrobial defense, proPO-activating cascade and redox, as described below.

#### Pattern recognition proteins

Invading microbes are detected by pattern recognition proteins (PRPs), which bind conserved pathogen-associated molecular patterns (PAMPs) shared by broad classes of microorganism. Several types of PRRs have been reported in invertebrates, such as peptidoglycan recognition proteins (PGRPs), β-1,3-Glucan recognition protein (βGRP), C-type lectins (CTLs), scavenger receptors (SRs), and thioester-containing proteins (TEPs) [25].

PGRP, first discovered in haemolymph of *B. mori*, binds bacterial peptidoglycan and triggers the prophenoloxidase cascade, culminating in the activation proPO and spätzle [26], [27]. PGRPs are considered to be the largest and most versatile family of pattern recognition molecules for microbial products in insects [28]. Indeed, we found that there are multiple putative *PGRP* unigenes in the house fly, each induced at all three time points post-infection, except that Unigene29467 was repressed at 6 h post-challenge. Of note, recent studies in *Drosophila* indicates that amidase PGRPs negatively regulates the IMD pathway by degrading PGN [29], [30], suggesting some late-expressed PGRPs in house fly may act to dampen immune response.

In contrast, *βGRP* was hardly responsive to infection, with only one gene (Unigene30021) mildly (1.54x) up-regulated at only one time point (6 h post-challenge). βGRPs were first identified in *B. mori* and crayfish *Pacifastacus leniusculus*, for their ability to bind β-glucans, and role in β-glucan-induced activation of phenoloxidase [31]. These proteins are found only in invertebrates and contain a protein domain that is similar to several bacterial glucanases. Some of the proteins have broader, some narrower defense specificities, and all are associated with the regulation of immune signaling pathways [32].

C-type lectins (CTLs) are a large family of PRRs found in almost all metazoans and mainly exert their functions depending on a common structural motif, the carbohydrate recognition domain [33]. The members of CTL family are abundant in shrimp and have various functions in innate immunity, including phagocytosis, melanization, respiratory burst, agglutination, antibacterial and anti-viral responses [34]. A total of 21 *CTL* unigenes were identified in the *M. domestica* transcriptome data, and most of them were massively up-regulated in larvae during various stages of infection. Thus, CLT may be important for antibacterial defense.

Scavenger receptors (SRs) recognize different PAMPs, including LPS, lipoteichoic acid (LTA) and yeast zymosan/β-glucan, and act as phagocytic receptors mediating non-opsonic phagocytosis of pathogens [35], [36]. We identified 73 unigenes 7 of them up-regulated at 48 h (the last stage post-infection) but none at earlier stages, raising the possibility that SRs might function to clean damaged biomolecules and even cell debris at late stage of infection.

Thioester-containing proteins (TEPs) are structurally related to the complement C3/alpha (2)-macroglobulin family [37], [38]. One of the best characterized TEPs in invertebrate is the mosquito *A. gambiae* TEP1, where upon bacterial infection, TEP1 was cleaved to release the C-terminal fragment that promotes phagocytosis of bacteria [39]. We found 40 putative unigenes, 6 of them up-regulated at 6 h and 48 h post-challenge, consistent with their roles in innate immunity.

#### Toll and IMD signaling pathways

Toll and IMD pathways activate antimicrobial peptide genes and regulate the host humoral response [40]. We identified many components in these two pathways, including *spätzle*, *toll*, *MyD*88, *tollip*, *tube*, *pellino*, *pelle*, *TRAF*2, *ECSIT*, *cactus*, *Dif*/*Dorsal*, *IMD*, *DREDD*, *TAK*1, *FADD*, *TAB*2, *IAP*2, *IKK* and *relish* (Table 2). A few unigenes encoding some of these proteins (e.g., *relish*, *cactus*, and *toll*) were up-regulated following bacterial infection with different expression patterns (Table S6).

#### Antimicrobial peptides

A hallmark of the insect defense is the induction and secretion of antimicrobial peptides that accumulate in the hemolymph where they oppose invading microorganisms [40]. Twenty unigenes encoding 4 known antimicrobial peptides were found to be up-regulated drastically in the infected *M. domestica* larvae at all three time points post-bacterial infection, reinforcing their crucial roles in innate immunity (Table S6), including *cecropin*, *attacin*, *defensin* and *diptericin*. We also found two novel dramatically up-regulated unigenes (Unigene29893 and Unigene13085), which we named *muscin* (GenBank: KF514663) and *domesticin* (GenBank: KF514664) (Figure S2 and Figure S3). The deduced peptides, containing putative signal peptides and rich in positively charged and hydrophobic amino acids, were synthesized and their antimicrobial activities tested using liquid growth inhibition assay. The two peptides were both active against all the tested bacteria (Table 4). Muscin and domesticin were most effective against *Serratia marcescens* and *Micrococcus luteus*, respectively (MIC 1.25–2.5 and 0.1–0.2 µM), and weaker activities were observed against *Salmonella typhimurium* (MIC 50–100 and 25–50 µM for muscin and domesticin respectively). However, the peptides lacked any detectable activity against fungi *Neurospora crassa* even at 100 µM. No homolog of muscin was found through searching the GenBank database,while domesticin shows some similarity to a few peptides of *Drosophila*, including IM18 peptide, an immune-induced molecule with unknown function (Figure S4) [41]. On the other hand, we failed to identify the homologus genes of *gloverin*, *moricin*, *lebocin*, antimicrobial peptides in *B. mori*. We predicted that the rapid evolution of antimicrobial peptide genes in insects is likely to be the result of host-pathogen co-evolution, indicating a specific role of antimicrobial peptides against a restricted subset of pathogens. Comparative analysis of gene repertoires of the antimicrobial peptides from different insects suggests that evolution of antimicrobial peptides followed independent scenarios as a result of specific adaptation to distinct ecological environments.

**Table 4:** Antibacterial activities of muscin and domesticin.

Microorganism	MIC (µM)	
	muscin	domesticin
**Gram-positive bacteria**		
*Bacillus megaterium*	2.5–5	0.2–0.4
*Bacillus* sp. DM-1	12.5–25	1.25–2.5
*Bacillus subtilis*	2.5–5	5–10
*Micrococcus luteus*	25–50	0.1–0.2
*Staphylococcus aureus*	12.5–25	2.5–5
**Gram-negative bacteria**		
*Agrobacterium tumefaciens*	25–50	5–10
*Escherichia coli* ATCC 25922	12.5–25	12.5–25
*Pseudomonas aeruginosa*	25–50	2.5–5
*Serratia marcescens*	1.25–2.5	0.5–1
*Salmonella typhimurium* CCTCCAB 94007	50–100	25–50
*Xanthomonas oryzae*	12.5–25	1.25–2.5
**Fungi**		
*Neurospora crassa*	>100	>100

MICs are expressed as the interval a–b, where a is the highest concentration tested at which microorganisms are growing and b the lowest concentration that causes 100% growth inhibition.

Antimicrobial peptide genes were dominant in up-regulated transcripts at all three time points post-bacterial infection. These results indicated that various antimicrobial peptides could respond rapidly to invaded pathogens and keep high-level expression during all these detected stages. The up-regulation of antimicrobial peptides expression after infection is a common defense strategy by hosts to rapidly destroy invaders. Differential expression patterns among different antimicrobial peptides may represent a combined strategy to form a defense network against diverse microbial pathogens.

Lysozymes, present in many organisms, cleave the β-1, 4-glycosidic linkage between N-acetylmuramic acid and N-acetylglucosamine found in certain bacterial cell walls [42]. Their functions have been widely described in species that ingest or harbour bacteria throughout their life cycles, such as *M. domestica* and *D. melanogaster*, and also characterized as digestive enzymes [42]–[45]. Other reports have suggested a major role of lysozymes in immune responses to pathogens [42], [46]. We found that two of the four *lysozyme* unigenes up-regulated and two other down-regulated post-infection, suggesting *Musca* lysozymes may function as defense molecules as well as digestive enzymes.

#### proPO-activating system

An immediate immune response in insects is the cell-mediated melanization reaction observed at the site of cuticular injury or on the surface of parasites invading the hemocoel. Melanization requires the activation of proPO (prophenoloxidase), an enzyme that catalyzes the oxidation of mono- and diphenols to orthoquinones, which polymerize nonenzy-matically to melanin [40]. The proPO-activating system mainly includes many genes such as serine proteinases and their inhibitors (*serpin*s), proPO-activating enzyme (*PPA*), *proPO* and its active form, phenoloxidase (*PO*) [47], [48]. After stimulated by injury or PAMPs, a serine proteinase cascade is first activated [49], which leads to the cleavage of the pro-form of the prophenoloxidase-activating enzyme (pro-PPA) into active PPA. Then PPA further activates the proPO into the active enzyme, PO, through proteolysis of its propeptide [47]. In the present study, many differentially expressed genes were annotated to be tentative members of the proPO-activating system (Table S6). These genes were mainly a kind of serine proteinases, and their inhibitors. The present gene expression data revealed that most members in the serine proteinase cascade and proPO system were responsive to bacterial infection in *M. domestica*. The expression level of *proPO* and *PPA* transcripts were increased significantly at 24 h or 48 h post-bacterial challenge. The expressions of most serine proteases were increased in bacterial-challenged larvae, indicating a positive response of the serine proteinase cascade in the immune defense. However, the profiles of *serpin*s were also up-regulated unexpectedly during this process, which seemed incompatible with their roles in regulation of the proPO-activating system. A similar consequence was shown in shrimp *Fenneropenaeus chinensis* [50]. It might be taken as a negative feedback mechanism to avoid damage of host tissues and cells by excess reactive components generated by PO.

#### Redox system

Reactive oxygen species (ROS) is referred to as a class of radical or non-radical oxygen-containing molecules that have high oxidative reactivity with lipids, proteins, and nucleic acids [51]. Generation of ROS in infected organisms not only helps to kill pathogens but also acts on the host cells themselves, including altering the intracellular redox balance and functioning as signaling molecules involved with the regulation of immunomodulatory genes. ROS production is regarded as an immediate acute-phase oxidative defense in response to pathogen assault or cellular stress such as phagocytosis and melanotic encapsulation [52]. Our analysis showed that the transcript levels of some cytochrome P450 and xanthine oxidase genes were strongly induced in the process of bacterial infection, especially at 48 h. Moreover, some unigenes encoding oxidoreductases, such as dehydrogenase/reductase SDR family member 11-like, 15-hydroxyprostaglandin dehydrogenaseor-related dehydrogenase, FAD-NAD binding oxidoreductase, peroxidasin-like, NADH dehydrogenase subunit 5 etc., were up-regulated significantly in this process. However, few unigenes encoding antioxidant enzymes were also seen up-regulations in this study. Previous study has revealed that *M. domestica SOD* genes could be induced mainly at 72 h post *E. coli* or *S. aureus* challenge [19]. We suppose that comprehensive up-regulated expressions of antioxidant enzyme genes would appear in the later stage. Furthermore, increasing sequencing depth in the future might increase both the number of immune related genes, and provide more data on their differential expression pre- and post-infection. There must be a fine redox balance maintained by innate immune system, which is critical for insect to survive from the war between pathogen and host. The underlying molecular mechanisms, however, still remain elusive. Our further research will focus on mitochondrion, an organelle which is considered as the central platform for innate immunity [53].

In addition to those known immune-related genes, we are surprised to find that *hexamerin*s are up-regulated strongly at 24 and 48 h post-challenge (Table S4). In general, hexamerin is thought to act as storage protein which is used as a source of amino acids and energy for protein synthesis during metamorphosis. But some reports indicated that insect hexamerin may be involved in innate immunity [54]–[56]. As humoral procoagulant, hexamerin can bind to the surfaces of bacteria invaded, and its subunits are confirmed as the major constituent of the aggregates in *Drosophila* hemolymph [54]. So we speculate that hexamerins might be involved in the innate immune response of the house fly.

### qPCR

To validate the gene expression result, qPCR analysis was performed using gene-specific primers for ten changed unigenes selected at random. Results are shown in Table 5 and are compared with the gene expression data. Although the qPCR data supports the trends of our gene expression results, the levels of change tend to be different between methods for each gene. We attribute differences in the level of change to the sensitivity of biases occurred between qPCR and gene expression. In general, the results of gene expression profiling are reliable.

**Table 5:** Comparisons of relative gene expression fold between gene expression data and qPCR results.

Name	gene ID	Fold change at 6/24/48 h (gene expression data)			qPCR fold change at 6/24/48 h		
*attacin*	Unigene12874	15.2	4.7	3.9	210.8±59.0**	52.6±15.8**	25.1±4.2**
*defensin*	CL7841.Contig1	15.9	3.4	6.0	220.3±60.5**	45.1±12.9**	63.2±16.1**
*diptericin*	Unigene13439	12.8	9.8	5.7	180.5±51.4**	112.3±30.8**	62.9±18.5**
*muscin*	Unigene29893	8.3	-	1.9	55.0±16.2**	16.0±4.3**	26.0±7.6**
*prophenoloxidase*	Unigene28739	-	-	10.9	42.3±12.1**	51.6±10.9**	116.7±32.2**
*PGRP-SD*	CL486.Contig1	4.2	1.4	1.6	7.8±2.4**	1.5±0.3**	1.1±0.3*
*HSP*67B2	CL8219.Contig1	-	1.1	1.2	1.0±0.3	0.9±0.2	1.0±0.2
*eiger*	CL358.Contig2	-	1.5	3.5	2.5±0.7**	3.9±1.1**	4.8±1.1**
*metallothionein*	Unigene13124	-	1.2	-	1.0±0.3	1.0±0.2	3.1±0.9**
*SOD*	CL6208.Contig1	-	-	1.9	1.9±0.5	1.1±0.3	2.1±0.5*

Significant differences between the challenged and control group are indicated with * at *P*<0.05 or ** at *P*<0.01.

## Conclusions

In this study, bacteria-challenged *M. domestica* transcriptome profiles were investigated and the substantial amount of transcripts was recovered, which provided a strong support for the future genomic research on *M. domestica*, especially on in-depth genome annotation in insects. Universally identified immune-candidate genes, infection markers, and putative signaling pathways were found in *M. domestica* and especially a considerable amount of immune-relevant genes and pathways in the house fly showed significant similarity to *Drosophila*, *Anopheles*, *Apis*, and *Bombyx*, suggesting that mechanisms underlying the innate immunity in insects might be conserved in invertebrates. After the bacterial challenge, pattern recognition proteins, especially the *PGRP*s, significantly increased. Antimicrobial peptides, including *ceropin*, *attacin*, *diptericin*, *defensin*, were also found increased. Moreover, component genes in Toll and IMD signaling pathways, as well as the genes involved in proPO-activating system and redox system, were strongly induced in the process of bacterial infection.

In addition, a large set of novel immune response genes that have never been linked previously to immune responses in other insects indicated that the immune system of insect might be much more complex than previously reported, and house fly-specific immune events might have happened during evolution as a result of specific adaptation to distinct ecological environments. Particularly, we realized that regulations of whole-body energy and metabolic homeostasis were disrupted, and there was a strong suppression of oxidative phosphorylation during antibacterial defense reaction. We firmly believe that unclear repair and rebalance mechanisms at the later stage of infection should be crucial for insect to survive from the pathogen-host battle.

Two novel antimicrobial peptides, *muscin* and *domesticin*, were found in challenged house flies, and they both showed broad spectrum of bactericidal activities. Domesticin is homologous to some peptides of *Drosophila*, including an immune-induced molecule IM18 peptide, while we failed to identify any known homologous peptide of muscin. This kind of newly found antimicrobial peptides could be a part of the explanation to how the house fly was able to flourish in the septic environments.

Although RNA-seq technology reduces the need of technical replication within our experiments and the fact that we used the NOISeq method can empirically model the noise in count data, the absence of biological replication within our experiments constrains the possibility of more detailed analyses. We feel safe to draw the draft conclusion above out of the multiple points data, including pre-challenge and post-challenge 6 h, 24 h, and 48 h. However, studies with more intensive time intervals and biological replications are needed to provide deep insight into the immunogenetics of the house fly, which also may contribute to a better understanding of the evolutionary history of innate immunity from insects to vertebrates.

## Supporting Information

Figure S1 **Length distribution of unigenes.**
(DOC)

Figure S2 **The nucleotide and deduced amino acid sequences of** ***M. domestica*** **antimicrobial peptide** ***muscin*** **.**
(DOC)

Figure S3 **The nucleotide and deduced amino acid sequences of** ***M. domestica*** **antimicrobial peptide** ***domsticin*** **.**
(DOC)

Figure S4 **Multiple alignment of domesticin with its homologs from** ***Drosophila*** **by CLC Sequence Viewer.**
(PDF)

Table S1 **Primers used for qPCR analysis.**
(DOC)

Table S2 **Top hits obtained by BLASTx for the unigenes.**
(XLS)

Table S3 **KO annotation of unigenes.**
(XLS)

Table S4 **The data of all the differentially expressed genes.**
(XLS)

Table S5 **KEGG enrichment analysis of all the differentially expressed genes.**
(XLSX)

Table S6 **Detail information of 509 putative immune-related differentially expressed genes.**
(XLS)

## References

[1] Hoffmann JA (2003) The immune response of *Drosophila* . Nature 426: 33–38. 14603309 10.1038/nature02021

[2] Tanaka H, Ishibashi J, Fujita K, Nakajima Y, Sagisaka A, et al (2008) A genome-wide analysis of genes and gene families involved in innate immunity of *Bombyx mori* . Insect Biochem Mol Biol 38: 1087–1110. 18835443 10.1016/j.ibmb.2008.09.001

[3] Graveley BR, Kaur A, Gunning D, Zipursky SL, Rowen L, et al (2004) The organization and evolution of the dipteran and hymenopteran Down syndrome cell adhesion molecule (Dscam) genes. RNA 10: 1499–1506. 15383675 10.1261/rna.7105504 PMC1370636

[4] Kocks C, Cho JH, Nehme N, Ulvila J, Pearson AM, et al (2005) Eater, a transmembrane protein mediating phagocytosis of bacterial pathogens in *Drosophila* . Cell 123: 335–346. 16239149 10.1016/j.cell.2005.08.034

[5] Royet J, Reichhart JM, Hoffmann JA (2005) Sensing and signaling during infection in *Drosophila* . Curr Opin Immunol 17: 11–17. 15653304 10.1016/j.coi.2004.12.002

[6] Kurata S, Ariki S, Kawabata S (2006) Recognition of pathogens and activation of immune responses in *Drosophila* and horseshoe crab innate immunity. Immunobiology 211: 237–249. 16697917 10.1016/j.imbio.2005.10.016

[7] Evans JD, Aronstein K, Chen YP, Hetru C, Imler JL, et al (2006) Immune pathways and defence mechanisms in honey bees *Apis mellifera* . Insect Mol Biol 15: 645–656. 17069638 10.1111/j.1365-2583.2006.00682.x PMC1847501

[8] Scott JG, Liu N, Kristensen M, Clark AG (2009) A case for sequencing the genome of *Musca domestica* (Diptera: Muscidae). J Med Entomol 46: 175–182. 19351068 10.1603/033.046.0202

[9] Guryev V, Cuppen E (2009) Next-generation sequencing approaches in genetic rodent model systems to study functional effects of human genetic variation. FEBS Lett 583: 1668–1673. 19379744 10.1016/j.febslet.2009.04.020

[10] Liu L, Li Y, Li S, Hu N, He Y, et al (2012) Comparison of next-generation sequencing systems. J Biomed Biotechnol 2012: 251364. 22829749 10.1155/2012/251364 PMC3398667

[11] Sackton TB, Lazzaro BP, Clark AG (2010) Genotype and gene expression associations with immune function in *Drosophila* . PLoS Genet 6: e1000797. 20066029 10.1371/journal.pgen.1000797 PMC2793509

[12] Grabherr MG, Haas BJ, Yassour M, Levin JZ, Thompson DA, et al (2011) Full-length transcriptome assembly from RNA-Seq data without a reference genome. Nat Biotechnol 29: 644–652. 21572440 10.1038/nbt.1883 PMC3571712

[13] Liu F, Tang T, Sun L, Jose Priya TA (2012) Transcriptomic analysis of the housefly (*Musca domestica*) larva using massively parallel pyrosequencing. Mol Biol Rep 39: 1927–1934. 21643958 10.1007/s11033-011-0939-3

[14] Iseli C, Jongeneel CV, Bucher P (1999) ESTScan: a program for detecting, evaluating, and reconstructing potential coding regions in EST sequences. Proc Int Conf Intell Syst Mol Biol 138–148. 10786296

[15] Li R, Yu C, Li Y, Lam TW, Yiu SM, et al (2009) SOAP2: an improved ultrafast tool for short read alignment. Bioinformatics 25: 1966–1967. 19497933 10.1093/bioinformatics/btp336

[16] Mortazavi A, Williams BA, McCue K, Schaeffer L, Wold B (2008) Mapping and quantifying mammalian transcriptomes by RNA-Seq. Nat Methods 5: 621–628. 18516045 10.1038/nmeth.1226 PMC13303166

[17] Tarazona S, Garcia-Alcalde F, Dopazo J, Ferrer A, Conesa A (2011) Differential expression in RNA-seq: a matter of depth. Genome Res 21: 2213–2223. 21903743 10.1101/gr.124321.111 PMC3227109

[18] Audic S, Claverie JM (1997) The significance of digital gene expression profiles. Genome Res 7: 986–995. 9331369 10.1101/gr.7.10.986

[19] Tang T, Huang DW, Zhou CQ, Li X, Xie QJ, et al (2012) Molecular cloning and expression patterns of copper/zinc superoxide dismutase and manganese superoxide dismutase in *Musca domestica* . Gene 505: 211–220. 22750315 10.1016/j.gene.2012.06.025

[20] Livak KJ, Schmittgen TD (2001) Analysis of relative gene expression data using real-time quantitative PCR and the 2(-Delta Delta C(T)) Method. Methods 25: 402–408. 11846609 10.1006/meth.2001.1262

[21] Swift M (1997) Graph Pad Prism, data analysis, and scientific graphing. Journal of Chemical Information & Computer Sciences 37: 411–412.

[22] Bulet P, Dimarcq JL, Hetru C, Lagueux M, Charlet M, et al (1993) A novel inducible antibacterial peptide of *Drosophila* carries an O-glycosylated substitution. J Biol Chem 268: 14893–14897. 8325867

[23] Casteels P, Ampe C, Jacobs F, Tempst P (1993) Functional and chemical characterization of Hymenoptaecin, an antibacterial polypeptide that is infection-inducible in the honeybee (*Apis mellifera*). J Biol Chem 268: 7044–7054. 8463238

[24] Zagrobelny M, Scheibye-Alsing K, Jensen NB, Moller BL, Gorodkin J, et al (2009) 454 pyrosequencing based transcriptome analysis of *Zygaena filipendulae* with focus on genes involved in biosynthesis of cyanogenic glucosides. BMC Genomics 10: 574. 19954531 10.1186/1471-2164-10-574 PMC2791780

[25] Yu XQ, Zhu YF, Ma C, Fabrick JA, Kanost MR (2002) Pattern recognition proteins in *Manduca sexta* plasma. Insect Biochem Mol Biol 32: 1287–1293. 12225919 10.1016/s0965-1748(02)00091-7

[26] Yoshida H, Kinoshita K, Ashida M (1996) Purification of a peptidoglycan recognition protein from hemolymph of the silkworm, *Bombyx mori* . J Biol Chem 271: 13854–13860. 8662762 10.1074/jbc.271.23.13854

[27] Jiang H, Vilcinskas A, Kanost MR (2010) Immunity in lepidopteran insects. Adv Exp Med Biol 708: 181–204. 21528699 10.1007/978-1-4419-8059-5_10 PMC9284565

[28] Royet J, Gupta D, Dziarski R (2011) Peptidoglycan recognition proteins: modulators of the microbiome and inflammation. Nat Rev Immunol 11: 837–851. 22076558 10.1038/nri3089

[29] Zaidman-Remy A, Herve M, Poidevin M, Pili-Floury S, Kim MS, et al (2006) The *Drosophila* amidase PGRP-LB modulates the immune response to bacterial infection. Immunity 24: 463–473. 16618604 10.1016/j.immuni.2006.02.012

[30] Bischoff V, Vignal C, Duvic B, Boneca IG, Hoffmann JA, et al (2006) Downregulation of the *Drosophila* immune response by peptidoglycan-recognition proteins SC1 and SC2. PLoS Pathog 2: e14. 16518472 10.1371/journal.ppat.0020014 PMC1383489

[31] Lee WJ, Lee JD, Kravchenko VV, Ulevitch RJ, Brey PT (1996) Purification and molecular cloning of an inducible gram-negative bacteria-binding protein from the silkworm, *Bombyx mori* . Proc Natl Acad Sci U S A 93: 7888–7893. 8755572 10.1073/pnas.93.15.7888 PMC38844

[32] Warr E, Das S, Dong Y, Dimopoulos G (2008) The Gram-negative bacteria-binding protein gene family: its role in the innate immune system of anopheles gambiae and in anti-Plasmodium defence. Insect Mol Biol 17: 39–51. 18237283 10.1111/j.1365-2583.2008.00778.x

[33] Robinson MJ, Sancho D, Slack EC, LeibundGut-Landmann S, Reis e Sousa C (2006) Myeloid C-type lectins in innate immunity. Nat Immunol 7: 1258–1265. 17110942 10.1038/ni1417

[34] Wang XW, Wang JX (2013) Pattern recognition receptors acting in innate immune system of shrimp against pathogen infections. Fish Shellfish Immunol 34: 981–989. 22960101 10.1016/j.fsi.2012.08.008

[35] Mukhopadhyay S, Gordon S (2004) The role of scavenger receptors in pathogen recognition and innate immunity. Immunobiology 209: 39–49. 15481139 10.1016/j.imbio.2004.02.004

[36] Areschoug T, Gordon S (2009) Scavenger receptors: role in innate immunity and microbial pathogenesis. Cell Microbiol 11: 1160–1169. 19388903 10.1111/j.1462-5822.2009.01326.x

[37] Bou Aoun R, Hetru C, Troxler L, Doucet D, Ferrandon D, et al (2011) Analysis of thioester-containing proteins during the innate immune response of *Drosophila melanogaster* . J Innate Immun 3: 52–64. 21063077 10.1159/000321554 PMC3031515

[38] Lagueux M, Perrodou E, Levashina EA, Capovilla M, Hoffmann JA (2000) Constitutive expression of a complement-like protein in toll and JAK gain-of-function mutants of *Drosophila* . Proc Natl Acad Sci U S A 97: 11427–11432. 11027343 10.1073/pnas.97.21.11427 PMC17216

[39] Levashina EA, Moita LF, Blandin S, Vriend G, Lagueux M, et al (2001) Conserved role of a complement-like protein in phagocytosis revealed by dsRNA knockout in cultured cells of the mosquito, *Anopheles gambiae* . Cell 104: 709–718. 11257225 10.1016/s0092-8674(01)00267-7

[40] Lemaitre B, Hoffmann J (2007) The host defense of *Drosophila melanogaster* . Annu Rev Immunol 25: 697–743. 17201680 10.1146/annurev.immunol.25.022106.141615

[41] Uttenweiler-Joseph S, Moniatte M, Lagueux M, Van Dorsselaer A, Hoffmann JA, et al (1998) Differential display of peptides induced during the immune response of *Drosophila*: a matrix-assisted laser desorption ionization time-of-flight mass spectrometry study. Proc Natl Acad Sci U S A 95: 11342–11347. 9736738 10.1073/pnas.95.19.11342 PMC21644

[42] Ursic Bedoya RJ, Mitzey AM, Obraztsova M, Lowenberger C (2005) Molecular cloning and transcriptional activation of lysozyme-encoding cDNAs in the mosquito *Aedes aegypti* . Insect Mol Biol 14: 89–94. 15663778 10.1111/j.1365-2583.2004.00534.x

[43] Regel R, Matioli SR, Terra WR (1998) Molecular adaptation of *Drosophila melanogaster* lysozymes to a digestive function. Insect Biochem Mol Biol 28: 309–319. 9692234 10.1016/s0965-1748(97)00108-2

[44] Nayduch D, Joyner C (2013) Expression of lysozyme in the life history of the house fly (*Musca domestica* l.). J Med Entomol 50: 847–852. 23926784 10.1603/me12167 PMC3947532

[45] Ito Y, Nakamura M, Hotani T, Imoto T (1995) Insect lysozyme from house fly (*Musca domestica*) larvae: possible digestive function based on sequence and enzymatic properties. J Biochem 118: 546–551. 8690715 10.1093/oxfordjournals.jbchem.a124943

[46] Gao Y, Fallon AM (2000) Immune activation upregulates lysozyme gene expression in *Aedes aegypti* mosquito cell culture. Insect Mol Biol 9: 553–558. 11122464 10.1046/j.1365-2583.2000.00216.x

[47] Cerenius L, Soderhall K (2004) The prophenoloxidase-activating system in invertebrates. Immunol Rev 198: 116–126. 15199959 10.1111/j.0105-2896.2004.00116.x

[48] Strand M (2008) The insect cellular immune response. Insect Science 15: 1–14.

[49] Jiang H, Kanost MR (2000) The clip-domain family of serine proteinases in arthropods. Insect Biochem Mol Biol 30: 95–105. 10696585 10.1016/s0965-1748(99)00113-7

[50] Li S, Zhang X, Sun Z, Li F, Xiang J (2013) Transcriptome analysis on Chinese shrimp *Fenneropenaeus chinensis* during WSSV acute infection. PLoS One 8: e58627. 23527000 10.1371/journal.pone.0058627 PMC3602427

[51] Morgan MJ, Liu ZG (2011) Crosstalk of reactive oxygen species and NF-kappaB signaling. Cell Res 21: 103–115. 21187859 10.1038/cr.2010.178 PMC3193400

[52] Ribou AC, Reinhardt K (2012) Reduced metabolic rate and oxygen radicals production in stored insect sperm. Proc Biol Sci 279: 2196–2203. 22279170 10.1098/rspb.2011.2422 PMC3321705

[53] Arnoult D, Soares F, Tattoli I, Girardin SE (2011) Mitochondria in innate immunity. EMBO Rep 12: 901–910. 21799518 10.1038/embor.2011.157 PMC3166463

[54] Wang Z, Wilhelmsson C, Hyrsl P, Loof TG, Dobes P, et al (2010) Pathogen entrapment by transglutaminase–a conserved early innate immune mechanism. PLoS Pathog 6: e1000763. 20169185 10.1371/journal.ppat.1000763 PMC2820530

[55] Beresford PJ, Basinski-Gray JM, Chiu JK, Chadwick JS, Aston WP (1997) Characterization of hemolytic and cytotoxic Gallysins: a relationship with arylphorins. Dev Comp Immunol 21: 253–266. 9258607 10.1016/s0145-305x(97)00011-6

[56] Freitak D, Wheat CW, Heckel DG, Vogel H (2007) Immune system responses and fitness costs associated with consumption of bacteria in larvae of *Trichoplusia ni* . BMC Biol 5: 56. 18154650 10.1186/1741-7007-5-56 PMC2235825

[57] Christophides GK, Zdobnov E, Barillas-Mury C, Birney E, Blandin S, et al (2002) Immunity-related genes and gene families in *Anopheles gambiae* . Science 298: 159–165. 12364793 10.1126/science.1077136

