# Supplementary material for: Transcriptional Response of Musca domestica Larvae to Bacterial Infection
Source: PLoS One. 2014 Aug 19;9(8):e104867. doi: 10.1371/journal.pone.0104867 (PMC4138075; doi:10.1371/journal.pone.0104867)
Supplement: Figure S1 — Length distribution of unigenes. (DOC) [file pone.0104867.s001.doc]

Figure S1. Length distribution of unigenes.
