# Supplementary material for: Transcriptional Response of Musca domestica Larvae to Bacterial Infection
Source: PLoS One. 2014 Aug 19;9(8):e104867. doi: 10.1371/journal.pone.0104867 (PMC4138075; doi:10.1371/journal.pone.0104867)
Supplement: Figure S2 — The nucleotide and deduced amino acid sequences of M. domestica antimicrobial peptide muscin . (DOC) [file pone.0104867.s002.doc]

3 CCA GAG TAA AAG CTG TGT GTC ATT CTT AAT CGA ACA GTA GGC GGA 47

48 TCG AAA CAA CAT CTA GAA TTG AAA TAA GAA ACA ATT AAT TAG AAA 92

93 ATG TTG GCA ATT CAT TCA TTA AGA AAT ATA CTC GTG GTG CTG CTA 137

0 Met Leu Ala Ile His Ser Leu Arg Asn Ile Leu Val Val Leu Leu 14

138 ATA TTC TCC CTT ATT CTG ACA GCG ATG GCG GAA TGG AAA CTA CCG 182

15 Ile Phe Ser Leu Ile Leu Thr Ala Met Ala Glu Trp Lys Leu Pro 29

183 GAT TTG ATT ATC AAC CAC ATT ACG CTA ACA CGA AGA AAT TGT TTT 227

30 Asp Leu Ile Ile Asn His Ile Thr Leu Thr Arg Arg Asn Cys Phe 44

228 AAA TAT CGA TGC GGC TAG ACC AGA GGA TTT GCA ATT CGA GAA AAA 272

45 Lys Tyr Arg Cys Gly End

273 TTT AGT CAA TAA GTT AAT TTA TTT TAT AAA AAA AAA AAC ATT AAT 317

318 AAC ATT TCC ATT TTA TAA AAA TAA TAA TAA ATA TGA CCC TTG AAA 362

363 AAC AAA AAA AAA AAA 377

Figure S2. The complete nucleotide and deduced amino acid sequences of *Musca domestica* antimicrobial peptide muscin. The signal peptide at the N-termini is underlined and shadowed letters (AATAAA) indicate the putative polyadenylation signal.
