# Supplementary material for: Transcriptional Response of Musca domestica Larvae to Bacterial Infection
Source: PLoS One. 2014 Aug 19;9(8):e104867. doi: 10.1371/journal.pone.0104867 (PMC4138075; doi:10.1371/journal.pone.0104867)
Supplement: Figure S3 — The nucleotide and deduced amino acid sequences of M. domestica antimicrobial peptide domsticin . (DOC) [file pone.0104867.s003.doc]

1 GAT TTT TTT CCT GTC TAC GAA TCT TTC TAT AAA ATG ATG CAG CCT 45

46 CAG AGA CAT CTT CAC AGT TAC TCA AAT CAT AAT CTT AGA GCA AAA 90

91 ACT CTT TAC TAG AAC TTC GAA AAT ATG AAA TTT TTC ACA CTC CTG 135

Met Lys Phe Phe Thr Leu Leu 7

136 GCT GCA CTG ATG GCA TTG TTT GCC ATC TGT AAT AAT TTC TCG ATG 180

8 Ala Ala Leu Met Ala Leu Phe Ala Ile Cys Asn Asn Phe Ser Met 22

181 GTA TCG GCA TCT CGT GAC TCC AGA CCT GTT CAA CCT AGA GTT CAA 225

23 Val Ser Ala Ser Arg Asp Ser Arg Pro Val Gln Pro Arg Val Gln 37

226 CCA CCA CCG CCA CCA CCC AAA CAA AAA CCA TTC ATC TAT GAT GCA 270

38 Pro Pro Pro Pro Pro Pro Lys Gln Lys Pro Phe Ile Tyr Asp Ala 52

271 CCA ATT AGA AGG CCA GGA GGA CGA AAA ACT ATG TAC GCT TAA AGT 315

53 Pro Ile Arg Arg Pro Gly Gly Arg Lys Thr Met Tyr Ala End

316 GTT TAT ATT TGT ACT TTT TAA TAA ATT TTA TTT ACG GCA AAA AAA 360

361 AAA 363

Figure S3. The complete nucleotide and deduced amino acid sequences of *Musca domestica* antimicrobial peptide domesticin. The signal peptide at the N-termini is underlined and shadowed letters (AATAAA) indicate the putative polyadenylation signal.
