# Supplementary material for: Transcriptional Response of Musca domestica Larvae to Bacterial Infection
Source: PLoS One. 2014 Aug 19;9(8):e104867. doi: 10.1371/journal.pone.0104867 (PMC4138075; doi:10.1371/journal.pone.0104867)
Supplement: Figure S4 — Multiple alignment of domesticin with its homologs from Drosophila by CLC Sequence Viewer. (PDF) [file pone.0104867.s004.pdf]

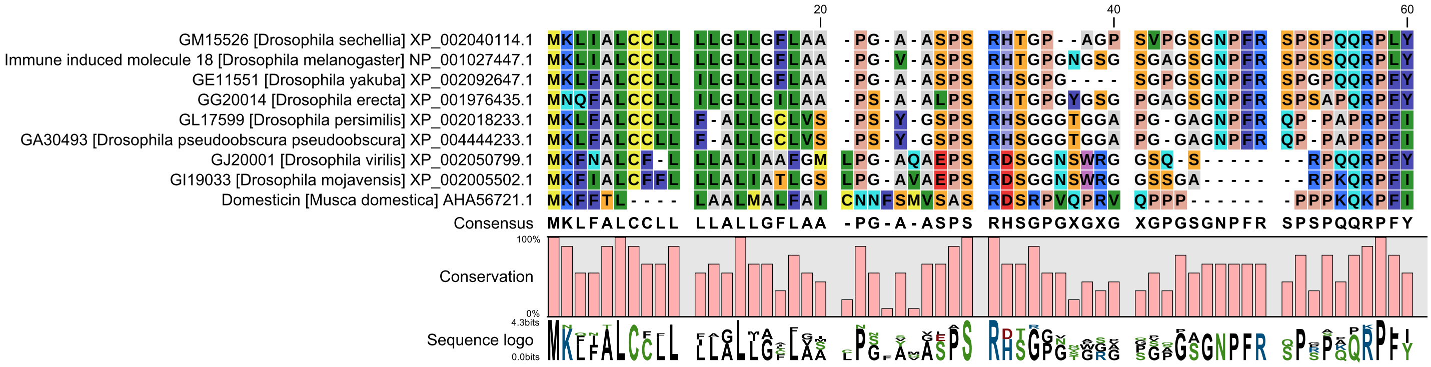

20
40
60
GM15526 [Drosophila sechellia] XP_002040114.1 MKLIALCCLL LLGLLGFLAA -PG-A-ASPS RHTGP--AGP SVPGSGNPFR SPSPQQRPLY
Immune induced molecule 18 [Drosophila melanogaster] NP_001027447.1 MKLIALCCLL LLGLLGFLAA -PG-V-ASPS RHTGPGNGSG SGAGSGNPFR SPSSQQRPLY
GE11551 [Drosophila yakuba] XP_002092647.1 MKLFALCCLL ILGLLGFLAA -PG-A-ASPS RHSGPG---- SGPGSGNPFR SPGPQQRPFY
GG20014 [Drosophila erecta] XP_001976435.1 MNQFALCCLL ILGLLGILAA -PS-A-ALPS RHTGPGYGSG PGAGSGNPFR SPSAPQRPFY
GL17599 [Drosophila persimilis] XP_002018233.1 MKLFALCCLL F-ALLGCLVS -PS-Y-GSPS RHSGGGTGGA PG-GAGNPFR QP-PAPRPFI
GA30493 [Drosophila pseudoobscura pseudoobscura] XP_004444233.1 MKLFALCCLL F-ALLGCLVS -PS-Y-GSPS RHSGGGTGGA PG-GAGNPFR QP-PAPRPFI
GJ20001 [Drosophila virilis] XP_002050799.1 MKFNALCF-L LLALIAAFGM LPG-AQAEPS RDSGGNSWRG GSQ-S----- --RPQQRPFY
GI19033 [Drosophila mojavensis] XP_002005502.1 MKFIALCFFL LLALIATLGS LPG-AVAEPS RDSGGNSWRG GSSGA----- --RPKQRPFI
Domesticin [Musca domestica] AHA56721.1 MKFFTL---- LAALMALFAI CNNFSMVSAS RDSRPVQPRV QPPP------ -PPPKQKPFI
Consensus MKLFALCCLL LLALLGFLAA -PG-A-ASPS RHSGPGXGXG XGPGSGNPFR SPSPQQRPFY
Conservation
100%
0%
Sequence logo
4.3bits
0.0bits

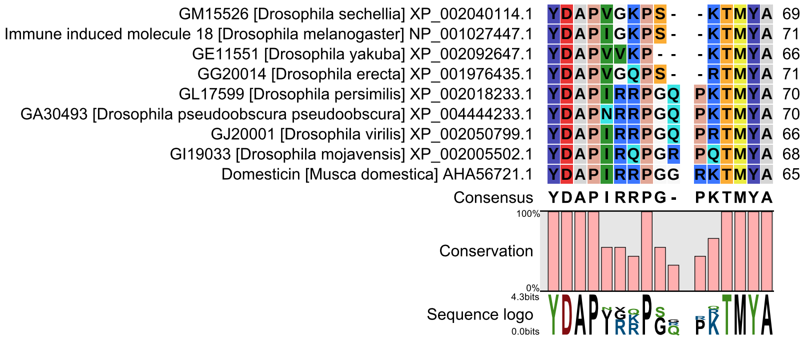
GM15526 [Drosophila sechellia] XP_002040114.1 YDAPVGKPS- -KTMYA 69
Immune induced molecule 18 [Drosophila melanogaster] NP_001027447.1 YDAPIGKPS- -KTMYA 71
GE11551 [Drosophila yakuba] XP_002092647.1 YDAPVVKP-- -KTMYA 66
GG20014 [Drosophila erecta] XP_001976435.1 YDAPVGQPS- -RTMYA 71
GL17599 [Drosophila persimilis] XP_002018233.1 YDAPIRRPGQ PKTMYA 70
GA30493 [Drosophila pseudoobscura pseudoobscura] XP_004444233.1 YDAPNRRPGQ PKTMYA 70
GJ20001 [Drosophila virilis] XP_002050799.1 YDAPIRRPGQ PRTMYA 66
GI19033 [Drosophila mojavensis] XP_002005502.1 YDAPIRQPGR PQTMYA 68
Domesticin [Musca domestica] AHA56721.1 YDAPIRRPGG RKTMYA 65
Consensus YDAPIRRPG- PKTMYA
Conservation
100%
0%
Sequence logo
4.3bits
0.0bits
