# Supplementary material for: Transcriptional Response of Musca domestica Larvae to Bacterial Infection
Source: PLoS One. 2014 Aug 19;9(8):e104867. doi: 10.1371/journal.pone.0104867 (PMC4138075; doi:10.1371/journal.pone.0104867)
Supplement: Table S1 — Primers used for qPCR analysis. (DOC) [file pone.0104867.s005.doc]

**Table S1. Primers used for qRT-**PCR analysis

| Gene | Primer Name | Sequence (5'-3') | Annealing temperature (°C) |
| --- | --- | --- | --- |
| β-actin | actinF | GAGAAATCCTATGAACTTCCCGACG | 62.0 |
|  | actinR | GGATACCGCAAGATTCCATACCCAA |  |
| attacin | attF | ATATTAGCGGCCACGAATCCC | 58.0 |
|  | attR | TTGGTCGTCAGTTTGGAAGCA |  |
| defensin | defF | ACACCTACCCACAAACCATCA | 57.2 |
|  | defR | TGTAACTCAGGCTCCAACTGC |  |
| diptericin | dipF | AATAGCCAAAGGCATTCTCCA | 59.6 |
|  | dipR | TGTGACTTATCGTCGGCAACC |  |
| muscin | musF | ATACTCGTGGTGCTGCTAAT | 57.8 |
|  | musR | CGCTCGAGATCGCAAATCCTCT |  |
| prophenoloxidase | pheF | CAACAACGCCTTACCGAACAG | 57.2 |
|  | pheR | CCATGACGCCGAATGACTCTA |  |
| PGRP-SD | pgrpF | CGAGGGTCGTGGTTGGAAGTA | 60.2 |
|  | pgrpR | TGGCTGGGCAGTTGGTTATTG |  |
| heat shock protein 67B2 | hspF | TTTTGGGCTCTGCCTCCTTTC | 59.2 |
|  | hspR | GCTTCAACTGGTGCCATACCG |  |
| eiger | eigF | GAATGAAGTTGAAACATCCAAG | 57.5 |
|  | eigR | GTACACATAATAAAGGCCCGGT |  |
| metallothionein | metF | GTTGTCCCTGTGACCAGCACT | 57.8 |
|  | metR | CACGTCCATCTTTGATTCATT |  |
| superoxide dismutase | sodF | TTGGAACCCATTGTCTGTCGG | 59.3 |
|  | sodR | CATTGAAACGCAAAGCAGGAG |  |
